# Remarkable Surgical Case, with Operation by Prof. Freer, Assisted by Prof. Powell, Cook County Hospital Clinic, May 10, 1872

**Published:** 1872-05

**Authors:** John E. O’Brien


ARTICLE IV. — *Remarkable Surgical Case, with Operation by Prof.* FREER, *assisted by Prof.* POWELL. *Cook County Hospital Clinic, May* 10, 1872. Reported by JOHN E. O'BRIEN, M.D.

HISTORY OF THE CASE.

The patient—Wm. Jansberg, aged nineteen; German; laborer; admitted May 7th—states that he has been subject to a swelling in the right iliac region at intervals for ten years; it occurred often, but he was always able to reduce it himself, except on two occasions, when he had to call a surgeon who was successful in reducing the tumor each time by taxis. Patient has worn a truss since last fall.

On the 6th inst. he was engaged in lifting a good deal all day, and riding home in a wagon afterwards, he suddenly found his tumor rising up more suddenly, in greater size, and with more tenderness than ever before; his efforts at reduction were futile, and on admission to the hospital he presented an irreducible tumor in the right iliac region, and his condition on the day of operation, as shown by Prof. Freer to the class, was as follows:

"This patient presents in the right iliac region a tumor of the size of a fœtal head, oblong in shape, its long diameter running from a point one inch above the centre of Poupart's ligament, outwards and upwards to a point two inches superior and internal to the anterior superior spinous process of the ileum; he is suffering from obstruction of the bowels, has vomited twice during the night, has the anxious features and hurried respiration and the great local tenderness peculiar to strangulated hernia. One important sign of complete strangulation he does not present, however, namely, the rapid pulse which usually attends it—his pulse counts only sixty beats per minute; we therefore conclude that the coil of intestine, which we here find compressed and irreducible in this unusual situation, is not yet completely strangulated.

"The question now arises as to what structure forms the stricture of this hernia: is it the internal abdominal ring? His right testicle has never descended through its inguinal canal; such a condition is usually considered a safeguard against hernia, and it is the rarest thing in the world to find the two, an undescended testicle and a hernia, incarcerated together in the inguinal canal.

"Suspend judgment for the present as to whether the hernia has escaped through the internal ring or not, and call to mind what other constriction a hernia in this high, unusual situation, might have. A knuckle of intestine may pass through some space in or between the abdominal muscles; the causes of such lesions being various, and the records of recovery after operation in such cases, being unfavorable. The books also speak of loops of intestine passing through the mesentery, and through false membranous bands, the product of peritonitis. Sometimes the vermiform appendix forms, by adhesion to some neighboring structure, a loop through which a knuckle of intestine may slip, and swell and become strangulated.

"This tumor is far removed from the location of an inguinal hernia, but what the cause of constriction is can only be demonstrated by an exploratory incision. One thing is certain, that the patient will die if we do not operate; and the longer we defer the operation, the more rapidly will the chances of successful operation diminish, for obvious reasons."

Observing that "we must be careful to stanch all hemorrhage as we proceed into the abdominal walls before opening the peritoneum," Prof. Freer made an incision, four inches long, upon the long axis of the tumor, viz., upward and outwards, dividing the skin and sub-cutaneous tissues, and exposing the aponeurosis of the external oblique; this, and the cellular tissue beneath, having been divided upon a director to the extent of the first incision, the slight hemorrhage which followed was arrested by torsion by Prof. Powell, and the pale semi-tendinous fibres of the internal oblique showed themselves. Remarking that "we must keep the relation of the epigastric artery (internal to our incisions) in view," Prof. Freer divided the internal oblique and the transversalis muscles upon a director, and the sac was exposed, lying in the region described, and upon the transversalis fascia, which it had bulged back into the abdomen as much as the muscles had been bulged forward. Opening the sac, a large quantity of serous accumulation escaped; the intestine was found congested but not in a stage of exudation; with it, was a large portion of omentum partly strangulated, and darkly congested, but showing no inflammatory exudate; and with these, a healthy-looking testicle, lying in the wound.

Tracing the back of the tumor downward and then backward, Prof. Freer found the stricture to consist of a hard, dense, contracted ring in the transversalis fascia, into which he succeeded in introducing first one forefinger and then another; all efforts failing to stretch the stricture sufficiently for the return of the intestine, Prof. Freer, with a probe-pointed bistoury, incised the upper edge of the ring, and Prof. Powell raising the truant intestine in his persuasive fingers its fluids gurgled back into the abdomen, whither it gradually followed.

The omentum was then carefully examined, and it was decided that it might be returned; the testicle was left in situ in the wound, and the latter neatly sewed up ,with seven stitches.

The hernia had escaped through an opening in the transversalis fascia formed by the testicle, in place of an inguinal canal, and worked its way upward between the transversalis fascia and muscle. This opening was no doubt started in fœtal life by the testicle resting against the transversalis fascia; where, finding no exit by the usual route, it worked a pouch upward, carrying with it the peritoneum as a *tunica vaginalis*, which pouch was afterwards filled and enlarged by the hernia and serous accumulation. The point of transversalis fascia upon which the testicle impinged may possibly have been the site of the internal ring, but Prof. Freer believes it to have been another and higher point. But this cannot be certainly determined as the patient is now, May 17th, recovering well from the operation.

